# Sympathoexcitation Associated with Renin-Angiotensin System in Metabolic Syndrome

**DOI:** 10.1155/2013/406897

**Published:** 2013-02-13

**Authors:** Takuya Kishi, Yoshitaka Hirooka

**Affiliations:** ^1^Department of Advanced Therapeutics for Cardiovascular Diseases, Kyushu University Graduate School of Medical Sciences, 3-1-1 Maidashi, Higashi-ku, Fukuoka 812-8582, Japan; ^2^Department of Advanced Cardiovascular Regulation and Therapeutics, Kyushu University Graduate School of Medical Sciences, 3-1-1 Maidashi, Higashi-ku, Fukuoka 812-8582, Japan

## Abstract

Renin-angiotensin system (RAS) is activated in metabolic syndrome (MetS), and RAS inhibitors are preferred for the treatments of hypertension with MetS. Although RAS activation is important for the therapeutic target, underlying sympathetic nervous system (SNS) activation is critically involved and should not be neglected in the pathogenesis of hypertension with MetS. In fact, previous studies have suggested that SNS activation has the interaction with RAS activation and/or insulin resistance. As a novel aspect connecting the importance of SNS and RAS activation, we and other investigators have recently demonstrated that angiotensin II type 1 receptor (AT_1_R) blockers (ARBs) improve SNS activation in patients with MetS. In the animal studies, SNS activation is regulated by the AT_1_R-induced oxidative stress in the brain. We have also demonstrated that orally administered ARBs cause sympathoinhibition independent of the depressor effects in dietary-induced hypertensive rats. Interestingly, these benefits on SNS activation of ARBs in clinical and animal studies are not class effects of ARBs. In conclusion, SNS activation associated with RAS activation in the brain should be the target of the treatment, and ARBs could have the potential benefit on SNS activation in patients with MetS.

## 1. Introduction

Metabolic syndrome (MetS) is characterized by visceral obesity, impaired fasting glucose, dyslipidemia, and hypertension [1, 2]. The increasing number of patients with MetS is a worldwide health problem because patients with MetS are considered to be at a high risk for cardiovascular disease. In the pathogenesis of MetS, renin-angiotensin system (RAS) is activated in various organs and tissues [3–6], and RAS inhibitors, such as angiotensin converting enzyme (ACE) inhibitors or angiotensin receptor blockers (ARBs), are preferred for the treatments of hypertension with MetS because of the prominent depressor effect with the improvement of insulin resistance [7–9]. Furthermore, in the pathogenesis of hypertension with MetS, underlying sympathetic nervous system (SNS) activation is critically involved [10–14], and previous studies have suggested that SNS activation has the interaction with insulin resistance [15] and/or RAS activation [16, 17]. In the animal studies, SNS activation is regulated by angiotensin-II-type-1-receptor-(AT_1_R-) induced oxidative stress in the brain [18–23], and recently, we have demonstrated that SNS activation is strongly mediated by AT_1_R-induced oxidative stress in the brain of animal models with MetS [24]. As the novel aspect connecting the importance of SNS and RAS activation, in the present paper, we focused on the SNS activation mediated by RAS activation in the brain of MetS.

## 2. Sympathetic Overactivation in MetS: Clinical Study

Insulin resistance and SNS activation have important roles in the pathogenesis of MetS [10, 15, 25–29]. Urinary excretion of catecholamine metabolites becomes elevated and more pronounced as the number of symptoms of MetS increases [27]. Sympathetic neural discharge is markedly potentiated [25], leading to increased insulin levels and elevated blood pressure [10]. Elevated levels of muscle sympathetic nerve activity (MSNA) are associated with obesity-induced subclinical organ damage, even in the absence of hypertension [30]. Interestingly, central obesity demonstrates augmented sympathetic outflow when compared to noncentral adiposity body types [27, 31–33] even when hypertension is not present. Furthermore, the presence of hypertension in MetS results in a further augmentation of the SNS activation [25, 33]. It should be noted that activation of the SNS is supposed to decrease the body weight. However, this does not occur in MetS with obese subjects. Recently, this is because of the interruption of the SNS activation as an action of energy expenditure suggested by Grassi [14] who modified the scheme originally made by Landsberg. Although it is difficult to prove this action in humans, activation of the brown adipose tissue, which increases energy expenditure, does not occur in obese subjects despite the fact that renal and lumbar SNS activation occur [34].

 The accumulation of body fat with a positive energy balance was first shown in animal models to result in SNS activation [35, 36]. The chronic increase in basal SNS activation is presumably aimed at stimulating *β*-adrenergic thermogenesis to prevent further fat storage [37] but can also stimulate lipolysis to increase nonesterified free fatty acids, contributing to insulin resistance. Adipose tissue itself can act as an endocrine organ and express various adipokines, which may directly or indirectly activate SNS [29]. A chronically elevated SNS activation could in turn impair *β*-adrenergic signaling, reduce stimulation of metabolism, and contribute to obesity and insulin resistance [10, 29]. Moreover, evidence demonstrates that insulin release increases MSNA and enhances the arterial baroreflex gain of SNS activation [38]. Furthermore, SNS activation is important for the occurrence and progression of hypertension leading to hypertensive organ damage in MetS [15]. Thus, treatments targeting the SNS activation are reasonable for patients with MetS.

## 3. Sympathetic Overactivation in Animal Models with MetS

It has been well documented that insulin can augment sympathetic outflow in animals via intracerebroventricular administration [39, 40]. Sympathetic outflow increases upon the injection of insulin into the third cerebral ventricle of rats [39]. A recent study also has demonstrated that the insulin affects arcuate nucleus, via the paraventricular nucleus of the hypothalamus, to increase the SNS activation and increase baroreflex gain of SNS activation [41]. While very little insulin is produced in the central nervous system, central insulin receptors are found on the hypothalamus [42] and can cause a coactivation of the SNS activation through transport-mediated uptake across the blood-brain barrier of peripherally secreted insulin [29]. In addition, the arcuate nucleus is unusual in that it contains highly permeable capillaries [43], such that insulin may directly activate receptors in this area without a specific transport mechanism [44]. These results suggest that the increase in plasma insulin causes sympathoexcitation via central mechanisms in animal models with MetS. As other mechanisms, we should discuss about leptin. Leptin is an adipocyte-derived hormone that has a key role in the regulation of the body weight through its actions on appetite and metabolism in addition to increasing blood pressure and SNS activation [40]. Rahmouni et al. suggested that mice with diet-induced obesity exhibit circulating hyperleptinemia and resistance to the metabolic actions of leptin. Recently, it was also demonstrated that RAS in the brain selectively facilitates renal and brown adipose tissue sympathetic nerve responses to leptin while sparing effects on food intake [45] and that hypothalamic arcuate nucleus plays an important role in mediating the sympathetic nerve responses to leptin and in the adverse sympathoexcitatory effects of leptin in obesity [46]. 

 In the other possible central mechanisms of sympathoexcitation in MetS, oxidative stress in the brain would be considered to play a pivotal role. Oxidative stress in the hypothalamus contributes to the progression of obesity-induced hypertension through central sympathoexcitation [47]. We also have demonstrated that AT_1_R-induced oxidative stress in the rostral ventrolateral medulla (RVLM) induces sympathoexcitation in rats with obesity-induced hypertension [24, 48]. RVLM is known as a major vasomotor center in the brainstem, and SNS activation is mediated by neuronal activity in the RVLM [49, 50]. In the RVLM, AT_1_R-induced oxidative stress has been determined to be a major sympathoexcitatory [21–23, 51]. Neurons in the RVLM contribute to elevated sympathetic outflow in rats with dietary-induced obesity [52]. In obesity-induced hypertension, systemic oxidative stress is increased and is associated with the development and progression of hypertension in various organs [53–56]. Taken together, it could be considered that SNS activation is increased in animal models with MetS via AT_1_R and oxidative stress in the brain.

## 4. Renin-Angiotensin System Activation in MetS

Previous many studies have demonstrated that RAS is activated in various organs and tissues in MetS [3–6, 29, 57]. Several peptides involved in the RAS have been implicated in insulin resistance [58–60] or hypertension [61, 62]. Hypercholesterolemia can increase AT_1_R gene expression on vascular smooth muscle cells [63, 64]. Low-density lipoprotein receptor-deficient mice fed a diet enriched in fat and cholesterol exhibited elevated plasma concentrations of angiotensinogen, angiotensin II [65], and brain angiotensinogen [66]. These results indicate that hypercholesterolemia stimulates the expression of several components of the RAS.

 Prolonged hyperglycemia and hyperinsulinemia could upregulate RAS [67–70]. Furthermore, angiotensin II can reduce whole body glucose utilization and insulin sensitivity, increase skeletal muscle and adipose tissue insulin resistance, and impair insulin signaling and action. Recent studies suggest that the RAS activation influences glucose homeostasis independent of its ability to regulate blood flow. Angiotensin II infusion into the interstitial space of skeletal muscle in dogs could result in insulin resistance independent of changes in blood flow [71]. Chronic angiotensin II infusion into insulin-sensitive rats was shown to reduce peripheral glucose use and insulin-induced glucose uptake [72]. In a model of angiotensin-II-induced hypertension, significant reduction in tyrosine phosphorylation of the insulin receptor and the insulin receptor substrate 1 in skeletal muscle was consistent with a whole-body reduction in insulin-mediated glucose transport [73]. Furthermore, RAS inhibitors could ameliorate insulin resistance [74, 75]. These studies could strongly suggest that RAS activation may contribute to insulin resistance in the MetS. Additionally, several large-scale clinical trials have demonstrated that the use of ARBs or ACE inhibitors can significantly reduce the incidence of new-onset diabetes in hypertensive patients and/or patients with MetS [76–79].

## 5. Renin-Angiotensin-System-Induced Sympathetic Overactivation in MetS

Both SNS and RAS are activated in obesity, and both systems can upregulate the action of the other [16, 17]. RAS is not only implicated in the observed sympathetic overdrive in obesity but may also provide a mechanism through which sympathetic overactivation leads to chronic hypertension [29]. In a previous clinical study, the inhibition of angiotensin II for three months in patients with MetS reduced MSNA activity by 21% [80]. 

 With regard to the central SNS regulation, sympathetic outflow is strongly mediated by RAS activation in the brain. It has already been demonstrated that RAS in the brain mediates SNS activation via oxidative stress in animal models with hypertension and/or heart failure [18–23]. In rats with obesity-induced hypertension, AT_1_R-induced oxidative stress in the RVLM induces sympathoexcitation [24, 48]. Taken together, it could be considered that SNS activation could be mediated by RAS activation and oxidative stress in the brain of MetS.

## 6. Angiotensin II Receptor Blockers Cause Sympathoinhibition in MetS 

In hypertensive patients with MetS, RAS inhibitors such as ACE inhibitors or ARBs are preferred [7–9]. In our recent study, we have found several new findings as follows: (1) telmisartan, but not candesartan, reduced plasma norepinephrine concentrations in the patients with MetS in spite of the similar depressor effects; (2) amelioration of baroreflex dysfunction in patients with MetS was significantly greater in the telmisartan-treated group than in the candesartan-treated group [28]. Our findings provide novel insight indicating that ARBs have beneficial effects on autonomic function in patients with MetS. Moreover, sympathoinhibitory effect of ARBs might not be a class effect. We also previously demonstrated that telmisartan inhibits SNS activation in hypertensive rats [22, 23]. In the animal studies, direct microinjection of ARBs into the RVLM or intracerebroventricular infusion of ARBs inhibits SNS activation in hypertensive rats [21, 81–83]. Interestingly, a previous study found that telmisartan can penetrate the blood-brain barrier in both a dose- and time-dependent manner to inhibit the centrally mediated effects of angiotensin II following peripheral administration [84]. We demonstrated that oxidative stress in the RVLM causes sympathoexcitation and baroreflex dysfunction [23, 51]. Taken together, these findings lead us to speculate that orally administered telmisartan, but not candesartan, could cause sympathoinhibition due to a reduction in the oxidative stress in the brain. Although other ARBs also inhibit the central actions of angiotensin II in the brain [84–89], these effects might differ depending on the pharmacokinetics and properties of each drug [84]. For example, in terms of agonist activity of peroxisome proliferator-activated-receptor-(PPAR-) gamma, a previous study suggested that orally administered rosiglitazone, PPAR-gamma agonist, promotes a central antihypertensive effect via upregulation of PPAR-gamma and alleviation of oxidative stress in the RVLM of spontaneously hypertensive rats [90]. Although both of telmisartan and candesartan have the function as a partial agonist of PPAR-gamma, only telmisartan can achieve this effect with therapeutics doses [91], and telmisartan might have benefits associated with agonistic effect of PPAR-gamma to a greater extent than candesartan [87, 88]. Further studies are necessary to clarify whether the ARBs-induced sympathoinhibitory effect is dependent on the central PPAR-gamma in MetS or not. 

## 7. Summary

**Figure 1:**
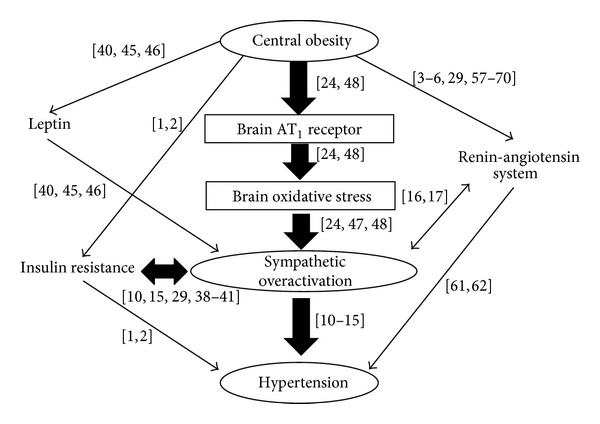
A schema presenting our concept in the regulation of sympathetic nervous system mediated by brain renin-angiotensin system in the metabolic syndrome. The numbers on the different arrows are the references from the bibliography.

RAS and SNS are abnormally activated in MetS, and there are interactions between RAS, insulin resistance, and SNS activation. Among these interactions, SNS activation is mainly augmented by RAS activation and oxidative stress in the brain (Figure 1). In patients with MetS, SNS activation mediated by RAS activation and oxidative stress in the brain should be the target of the treatments for hypertension, and ARBs could have the potential benefit on SNS activation.
